# Lining Cavity Margins with Non-Cohesive Gold

**Published:** 1906-08

**Authors:** W. E. Driscoll

**Affiliations:** Bradentown, Fla.


### LINING CAVITY MARGINS WITH NON-COHESIVE GOLD.

By W. E. Driscoll, Bradentown, Fla.

(Florida State Dental Society, 1906.)

There seems to be far too much indifference among dentists as to the claims of non-cohesive gold over cohesive for preserving teeth. I shall not take the time to repeat these claims as they can be found in our text-books and periodical literature.

Nor shall I now describe the usual method of proceedure in filling either with non-cohesive gold alone or in combination with cohesive.

At a recent meeting of the society I advocated the use of more gold and less amalgam in filling teeth, and my present purpose is to further that idea so far as I can, believing that our patients can be greatly benefited by such an advance in practice, and at the same time our compensation be justly and materially augmented.

I hold that only a small proportion of cavities can be filled satisfactorily with non-cohesive gold alone. The proportion can be increased by excavating shallow cavities to a depth two or three times deeper than is necessary with cohesive gold alone, or with non-cohesive and cohesive properly combined. Dentine is generally too sensitive to justify this extra excavation, and one must be an expert in the use of non-cohesive gold to justify the sacrifice of so much tooth structure even where sensitiveness is not involved, for be it remembered, that unless non-cohesive gold is inserted as it should be it will fail as readily as the harder filling.

Since "Extension for prevention" has gained so many converts, any plan of filling teeth must or should make some concession to this practice, or show how the two may be harmonized, or reconciled and combined. This is necessary because extension for prevention removes the greater part of the retension walls upon which non-cohesive gold filling must depend for stability, but a combination of non-cohesive gold with cohesive is practicable in such cavities by the plan I will try to make plain in this paper. I will say, I hope it is by no means original in my practice. I can hardly believe it is, since it so naturally suggests itself to any one who becomes a convert to the importance of a combination of the advantages of the two preparations of gold for filling teeth.

Now I will describe the plan I practice. I prepare the cavity just as I would for cohesive gold alone, that is by removing all defective tooth structure back to such as is sound and firm. I use a combination of the groove and retaining point where the form of the cavity requires them for retaining the filling. I also bevel the margins of enamel slightly but carefully. I fill the retaining points and grooves and cavity with cohesive gold until I have only a small groove formed by the cohesive gold and the margin of the cavity all around the filling. In this groove I lay Pack's No. 1 or 2 non-cohesive cylinders, previously flattened and doubled to-gether once or twice, to make them narrow enough to nicely fill the groove and also extend slightly outside the enamel margin.

Before condensing one of these flatulated cylinders I lay a strip of No. 30 cohesive gold over it and extending inward so as to cohere with the central body of cohesive gold already described. The first pressure on these two layers of gold is made with a Varney No. 8, foot plugger, until only enough cohesive gold has been used to hold all firm. Then I use one of Dr. Head's No. 2 or 3 pluggers to drive the two kinds of gold down into the groove, but still allowing the non-cohesive gold to extend entirely over the enamel margin so as to secure a non-cohesive lining of that margin. This last plugger has a very narrow face, and is sometimes called a fissure filling. Each cylinder must be secured as described before another is placed. After the groove is filled, the remainder of the filling is made with cohesive gold just as if non-cohesive gold had been used.

I do not claim that this is the only, or best way of securing a non-cohesive gold lining for cavity margins, but trust my description may be of value to some who find a lack of permanence in many non-cohesive gold fillings. I have found that this plan is not applicable to contour filings in thin incisor teeth except at the cervix. The strain of biting is too great for permanence.

Since I have excepted thin incisors as not suited for this plan except at the cervex, I feel like stating my procedure with them though not coming under the title of this paper, strictly speaking.

To conceal as much gold as practicable I retain as much of the enamel in front as I can, and far more than would be possible without a good cement lining to support the thin plate of enamel. Instead of drilling retaining points in this cement for retaining the gold superstructure, I have three or more gold cylinders ready prepared. These may be of either cohesive or non-cohesive gold. I prefer Pack's No. 1 non-cohesive cylinders rolled rather tightly between the thumb and fingers. I fill the cavity full of cement and that will give me time before it sets to press into it at least three of the cylinders, going to the bottom of the cavity with one end and allowing the other to project to the surface of the cement. Just before the cement sets I press all flat with the surface of the enamel walls with a burnisher or spatula. When cement is hard enough, which should be only about five minutes, I push a small smooth retaining point filler to the bottom of each of these cylinders. In case I fail to get the point inserted sufficiently for a good retaining point, I use a No. 2 bur to help open a retaining point through the cylinder. Observation has convinced me that a better retaining point is secured in this way than to drill into the cement after it has set, and that a better lining of the cavity is also gained.

Please note that I have referred to this plan of cement lining because the question might arise as to what is my practice with thin incisors where the plan of lining the margins with non-cohesive gold is impracticable.

